# Site-Specific Antibody Conjugation for ADC and Beyond

**DOI:** 10.3390/biomedicines5040064

**Published:** 2017-11-09

**Authors:** Qun Zhou

**Affiliations:** Protein Engineering, Biologics Research, Sanofi, Framingham, MA 01701, USA; qun.zhou@genzyme.com

**Keywords:** site-specific conjugation, specific amino acids, unnatural amino acids, glycans, short peptide tags, ADC, other applications

## Abstract

Antibody-drug conjugates (ADCs) have become a promising class of antitumor agents with four conjugates being approved by regulatory agencies for treating cancer patients. To improve the conventional conjugations that are currently applied to generate these heterogeneous products, various site-specific approaches have been developed. These methods couple cytotoxins or chemotherapeutic drugs to specifically defined sites in antibody molecules including cysteine, glutamine, unnatural amino acids, short peptide tags, and glycans. The ADCs produced showed high homogeneity, increased therapeutic index, and strong antitumor activities in vitro and in vivo. Moreover, there are recent trends in using these next generation technologies beyond the cytotoxin-conjugated ADC. These site-specific conjugations have been applied for the generation of many different immunoconjugates including bispecific Fab or small molecule–antibody conjugates, immunosuppressive antibodies, and antibody–antibiotic conjugates. Thus, it is likely that additional technologies and related site-specific conjugates will emerge in the near future, with various chemicals or small molecular weight proteins in addition to cytotoxin for better treatment of many challenging diseases.

## 1. Introduction

Therapeutic antibodies have become a major class of biologics in treating many challenging diseases, including cancer. Due to the fact that they have high specificity and affinity, the monoclonal antibodies have less off-target side-effects as compared to small molecular weight compounds. Currently, many monoclonal antibodies are under accelerated clinical developments [1]. It was predicted that by 2020 there will be about 70 of them being approved by regulatory agencies and used in clinics in treating patients [2]. Besides the traditional immunoglobulin G (IgG), many different antibody formats are being developed to enhance therapeutic efficacy, such as antibody-drug conjugates (ADCs) and bispecific antibodies [3,4,5,6,7,8,9,10,11]. The ADCs contain both antibody and coupled cytotoxin or chemotherapeutic drug. They combine the advantages of antibodies with high affinity and specificity for cell surface antigens with small molecular drugs with high penetration towards intracellular targets for inducing apoptosis.

As one of the targeted therapies or the “magic bullet” proposed by Paul Ehrlich more than a century ago [12], the concept of ADCs is the targeted delivery of a highly cytotoxic drug for selective (as opposed to systemic) chemotherapy, resulting in improved therapeutic index and enhanced efficacy relative to traditional chemotherapies or naked monoclonal antibodies.

**Figure 1:**
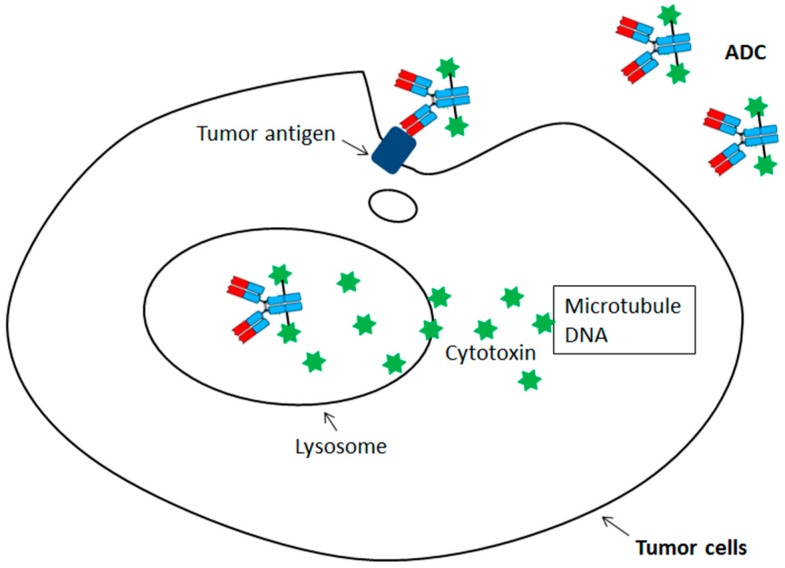
Binding and internalization of antibody-drug conjugate (ADC) followed by the release of its cytotoxins inside the cell.

Thus, this unique format would allow the use of certain chemotherapeutic drugs which are too potent or toxic to be applied systemically. Since these cytotoxins need to be delivered into the tumor cells targeting intracellular microtubules or DNA, the chemotherapeutic drugs carrying antibodies should be efficiently internalized into lysosome once binding to the antigen on the tumor surface (Figure 1). Within the lysosome, the cytotoxins would be efficiently released from antibodies through proteolytic cleavage or disulfide reduction before they diffuse into cytoplasm for cytotoxicities. Although the ADC idea has been around for decades, the medicine used in clinics was not available until 2000 with the regulatory approval of first ADC, gemtuzumab ozogamicin (anti-CD33 ADC). Currently, there are four ADCs approved by regulatory agencies for cancer treatment, including gemtuzumab ozogamicin (anti-CD33 ADC) for acute myelogenous leukemia (AML), brentuximab vedotin (anti-CD30 ADC) for treating anaplastic large cell lymphoma/Hodgkin’s lymphoma, trastuzumab emtansine (Anti-HER2 ADC) for advanced HER2 (human epidermal growth factor receptor 2)-positive breast cancer, and inotuzumab ozogamicin (anti-CD22 ADC) for treatment of relapsed or refractory acute lymphoblastic leukemia (ALL) [13,14,15,16,17,18,19,20,21,22].

All these ADCs were prepared using conventional conjugation methods that couple antibodies through either surface-exposed lysines (~70 to 90) or cysteines from interchain disulfides (8 in IgG1) after partial reduction. These lysine and cysteine methods generate heterogeneous products with varied numbers of drugs coupled across several possible sites although the average drug-to-antibody ratio (DAR) appears around 4 to 6, creating significant challenges for process consistency and product characterization [23,24,25]. It is also limited in understanding the relationships between the site/extent of drug loading and ADC attributes such as efficacy, safety, pharmacokinetics, and immunogenicity. The non-specific conjugation would also result in more off-target side-effects, leading to relatively low maximum tolerated dose. To improve the technology aiming for homogeneous molecule with higher therapeutic index, several site-specific ADC technologies have been developed as next generation methods [26].

**Figure 2:**
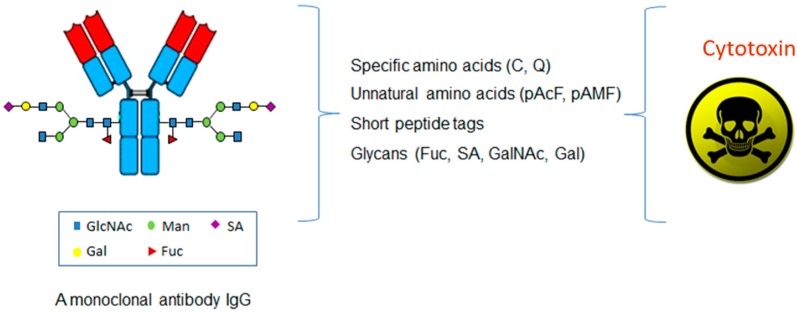
The categories of the site-specific ADC with cytotoxin coupled at unique and defined sites in an antibody molecule.

In contrast to the conventional conjugations to the lysines or cysteines, which are abundant in an antibody, the site-specific conjugations couple the antibodies with cytotoxins through the unique and defined sites based on antibody engineering. There are four major categories of methods based on the conjugation sites in the antibody molecules, including specific amino acids, unnatural amino acids, short peptide tags, or glycans (Figure 2 and Table 1).

## 2. Site-Specific ADC through Specific Amino Acids

Several native or engineered amino acids, including cysteines and glutamines, are selected as the sites for conjugation.

The unpaired cysteine-mediated conjugation was the first described site-specific ADC as THIOMAB or TDC [27]. A cysteine residue was engineered into different positions of antibody heavy chain (HC) or light chain (LC) for coupling. Due to the fact that the engineered cysteines are always capped with glutathione or something else during expression, the antibodies need to be partially reduced to remove the cap. The uncapped cysteines were then coupled with a thiol-reactive linkers containing cytotoxins, such as monomethyl auristatin E (MMAE), using thiol-maleimide chemistry. The ADCs generated through drug-linker conjugation at the cysteine residue (HC-A114C) showed nearly homogeneous conjugates with improved therapeutic index. The anti-MUC16 TDC displayed two-fold improved in vivo efficacy in a mouse xenograft model of ovarian cancer over the same MMAE containing ADC prepared using the conventional cysteine method at equivalent drug (or cytotoxin) dose, while both TDC (DAR at 1.6) and conventional ADC (DAR at 3.1) had similar efficacy at equivalent antibody dose. The TDC was tolerated at higher dose in rats and cynomolgus monkeys than the conventional ADC. Similar results were obtained with anti-HER2 TDC prepared with another tubulin inhibitor containing drug-linker, mertansine (DM1), as compared to the same DM1 containing ADC prepared using conventional lysine method [28]. In a safety study with cynomolgus monkey, the TDC was tolerated at higher antibody dose than the conventional ADC (48 mg/kg vs. 30 mg/kg). The THIOMAB approach was also applied to other antibodies, such as anti-CD70, with cytotoxic DNA cross-linking pyrrolobenzodiazepine (PBD) linker coupled at an engineered cysteine (S239C) in heavy chain (HC) [29]. The TDC showed low aggregation, high stability in plasma, and strong in vivo and in vitro antitumor activities.

Moreover, cysteine insertion was also described in antibody before and after selected sites in either IgG HC or LC, including LC-V205, HC-A114, and HC-S239 [30]. There was no difference in conjugation efficiency between cysteine-inserted and cysteine-substituted antibodies in coupling to a PBD drug-linker. The ADCs prepared with drug-linker coupled through the cysteine-insertion after site HC-S239 of anti-5T4 (trophoblast glycoprotein, an oncofetal antigen on breast tumor) demonstrated potent dose-dependent antitumor activity in a mouse xenograft model [30].

In addition to conjugation through unpaired cysteine, thiol bridge methods were developed to conjugate a bifunctional drug-linker to both cysteines from each of interchain disulfide, instead of only one cysteine in the conventional cysteine conjugation, after partial reduction and re-oxidation [31,32]. Thus, four drugs were coupled to eight cysteines from four interchain disulfides in IgG1 with low heterogeneity. In a method developed, a reduction bis-alkylation approach was applied to rebridge the reduced interchain disulfide bonds in antibody [31]. Anti-HER2 was conjugated with bisAlk-vc-MMAE with DAR at 4 as a major product. There was improved stability of the ADC prepared using this approach as compared to the ADC prepared using the conventional cysteine method. The ADC also showed higher in vivo antitumor efficacy than trastuzumab-DM1 prepared using conventional lysine approach. Behrens et al. reported the conjugation of the antibody with a bifunctional dibromomaleimide (DBM) linker instead of a conventional maleimide linker [32]. They found ~70% of DBM-MMAF derivative crosslinked both cysteines from interchain disulfides, while 30% of the drug-linker was half antibody conjugate due to intrachain crosslinking of cysteines originally from interchain disulfides. The ADCs demonstrated improved pharmacokinetics and reduced toxicity in vivo compared to analogous conventional cysteine ADCs. Anti-HER2 ADC prepared using this methods showed better in vivo efficacy than the ADC generated using conventional cysteine conjugation. It significantly delayed the tumor growth, while treatment with the conventional ADC did not result in any significant inhibition at similar dose in a mouse xenograft model of ovarian cancer.

The site-specific conjugation through glutamine was also reported [33,34]. Instead of using reducing and oxidizing reagents, the method was designed by using microbial transglutaminase (mTG) to transfer an amine containing drug-linker or a reactive spacer into HC-Q295 in a deglycosylated antibody. The conjugation was optimized using a two-step chemoenzymatic approach whereby a reactive spacer containing a bioorthogonal azido or thiol functional linker was attached to the antibody by mTG and subsequently reacted with either dibenzocyclooctynes (DBCO) or maleimide containing MMAE. By using strain-promoted azide-alkyne cycloaddition (SPAAC) or thiol-maleimide chemistry, homogeneous ADCs were generated with DAR at ~2. The Anti-HER2-MMAE showed strong in vitro potency against tumor cells.

The site-specific conjugation through unpaired cysteine is relatively simple and scalable. The drug coupling was done without the need of special reagents. The method has been applied to prepare multiple site-specific ADCs for preclinical or clinical developments. As described above, the ADCs prepared through site-specific cysteines showed two-fold stronger in vivo antitumor activities and were better tolerated than the conventional conjugates. However, the stability of conjugate generated through unpaired cysteine varied dependent on where the cysteine was introduced in an antibody molecule [35]. It appears that the TDC prepared with conjugation at highly solvent-accessible site in a relatively neutral environment (HC-S396C) rapidly lost the drug in plasma due to maleimide exchange with other reactive thiols, such as albumin, glutathione or cysteine present in plasma. The TDC prepared by conjugating drug-linker to a partially accessible site with a positively charged environment (LC-V205C) showed the least thiol maleimide exchange, while the conjugate at the site with partial solvent-accessibility and neutral charge (HC-A114C) displayed the intermediate drug stability in plasma.

## 3. Site-Specific ADC through Unnatural Amino Acids

The coupling of drug-linker to unnatural amino acid residues in the antibody is another approach for site-specific conjugation [36]. An orthogonal amber suppressor tRNA/aminoacyl-tRNA synthetase (aaRS) pair from *Methanococcus jannaschii* was expressed in the cells to site-specifically incorporate *p*-acetylphenylalanine (pAcF) in response to an amber nonsense codon engineered in the antibodies. The keto group of pAcF was then selectively coupled to a drug-linker with aminooxy functionality. The pAcF-containing anti-HER2 was produced from Chinese hamster ovary (CHO) cells co-expressing antibody with an amber codon at heavy chain (HC-A121) and an orthogonal amber suppressor tRNA/aaRS pair. The pAcF residue was coupled with a non-cleavable auristatin linker, which is a potent tubulin inhibitor, through oxime chemistry with DAR at ~2. The anti-HER2 ADC showed strong in vitro and in vivo antitumor activities. The method was further optimized, and a stable CHO cell line was generated to express orthogonal amber suppressor tRNA/aaRS pair [37]. Multiple pAcF containing antibodies, including anti-5T4 (trophoblast glycoprotein), anti-EGFR (epidermal growth factor receptor), anti-HER2, and anti-PSMA (prostate specific membrane antigen), were expressed in the cell line with titers over 1 g/L in fed-batch processes. The unnatural amino acid was introduced into different sites in these antibodies, such as HC-S115 for anti-5T4 or HC-A114 for anti-HER2. It was then conjugated with the drug-linker, aminooxy containing monomethyl auristatin D (MMAD), with DAR at ~2. Interestingly, the ADC prepared with this site-specific technology showed superior in vivo antitumor efficacy compared to the ADC prepared using either conventional conjugation through hinge disulfides or site-specific conjugation through unpaired cysteine as described above. Although all three ADCs caused complete regression of tumor growth at 3 mg/kg dose, only anti-HER2 HC-A114pAcF ADC showed complete tumor regression at 1 mg/kg in a moues tumor xenografts established from breast cancer cell lines [37,38].

CHO cells expressing the pyrrolysyl-tRNA synthetase (pylRS) and its cognate tRNA (tRNA pyl) were also generated to genetically encode an unnatural amino acid containing an azido moiety in response to an amber stop codon [39]. Anti-HER2 antibodies were expressed from the engineered cells to contain N6-((2-azidoethoxy)carbonyl)-l-lysine at four different positions in either heavy or light chain for DAR at 2 and combination of two sites in both heavy and light chains for DAR at 4. The azido group introduced at position HC-H274 of the antibody enabled click cycloaddition chemistry that generated a stable heterocyclic triazole linkage of the toxin auristatin F or PBD with over 95% conjugation efficiency. The ADCs were potent and specific in in vitro cytotoxicity assays. They demonstrated stability in vivo and a PBD containing ADC with DAR of 1.8 showed similar efficacy in sustained regression of tumor growth in a mouse tumor xenograft model as compared to the ADC with DAR of 3 prepared using conventional cysteine conjugation.

In addition, a cell-free expression system was established to produce ADCs through site-specific incorporation of the optimized unnatural amino acid, *p*-azidomethyl-l-phenylalanine (pAMF) [40]. A novel variant of the *Methanococcus jannaschii* tyrosyl tRNA synthetase (TyrRS) was discovered through library screening with a high activity and specificity toward pAMF. The site-specific incorporation of pAMF at HC-S136 of anti-HER2 facilitated near complete conjugation of a DBCO-PEG-monomethyl auristatin F (DBCO-PEG-MMAF) through SPAAC using copper-free click chemistry. The resultant ADCs showed in vitro antitumor potency.

The ADC was also site-specifically generated using selenocysteine (Sec) residues engineered at C-terminus of antibody with iodoacetamide containing monomethyl auristatin F (MMAF) [41]. In eukaryotes, Sec is encoded by the stop codon UGA, and its translational incorporation requires the presence of a Sec incorporation sequence (SECIS) in the UTR of the mRNA. Since the selenol group of Sec is more nucleophilic than the thiol group of Cysteine, the antibody was conjugated under mildly acidic and reducing conditions without antibody re-oxidation required for THIOMAB conjugation. The ADC showed strong antitumor activities in vitro and in vivo. Significant tumor regression and growth inhibition were observed for anti-HER2 scFv-Fc-Sec conjugate. Four of the five mice treated with the ADC at high dose were tumor free at six weeks after the last treatment.

The ADCs prepared through conjugation of drug-linkers to the unnatural amino acids were more efficacious in vivo than the conjugates generated using conventional or THIOMAB methods. The unnatural amino acid–containing antibodies were expressed in a bioreactor at gram scale, and the conjugates were stable. However, cell line engineering is required for optimal expression of orthogonal amber suppressor tRNA/aaRS pair in addition to antibody engineering. The potential immunogenicity of these unnatural amino acids containing antibody in human is currently unknown.

## 4. Site-Specific ADC through Glycans

The glycan-mediated conjugation provides a unique site-specific method by conjugating the drug-linker to N297 glycans located in CH2 domain instead of coupling the relatively hydrophobic cytotoxin into amino acid residues. Since there are several different monosaccharides present at non-reducing terminus of the glycans, various approaches were developed to conjugate the drug-linkers to these sugars, including fucose, galactose, *N*-acetylgalactosamine (GalNAc), *N*-acetylglucosamine (GlcNAc), and sialic acid (SA). It was reported by Okeley et al. that 6-thiofucose, a fucose analogue, could be metabolically incorporated into anti-CD30 or anti-CD70 antibodies. The thiofucose in the antibodies was then conjugated with maleimide containing MMAE drug-linker with DAR at ~1.3 [42]. The ADCs generated through thiofucose maintained good plasma stability and showed strong in vitro antitumor activity.

The galactose or GalNAc analogues were also introduced in vitro by a mutant galactosyltransferase, GalT (Y289L) which was discovered by Qasba et al. [43,44], for preparing site-specific ADCs with DAR close to 2. The ADC, which was generated using C-2 keto Galactose labeling and aminooxy containing auristatin F, showed strong in vitro antitumor potency [45]. The same enzyme, GalT (Y289L), was also used to introduce azido-GalNAc to core GlcNAc exposed after pre-treatment of antibodies with endoglycosidases, such as endo F3, endo S, or endo S2, which release most of N-297 glycans except for the innermost GlcNAc [46]. The drug-linkers, such as bicyclononyne (BCN) containing MMAE, MMAF, maytansine, or doxorubicin, were efficiently conjugated to the introduced azido-GalNAc through SPAAC using copper-free click chemistry. The process was scaled up for preparing 5 g ADCs with excellent homogeneity. The anti-HER2 ADCs prepared using this method with BCN containing maytansine or MMAF showed better in vivo antitumor efficacy than the anti-HER2 coupled with DM1 using a conventional lysine conjugation approach. In a breast cancer xenograft mouse model, anti-HER2 glycoconjugate containing a cleavable or noncleavable maytansine linker demonstrated complete tumor regression, while both the MMAE containing glycoconjugate and conventional lysine conjugate showed partial tumor suppression.

Moreover, sialic acid (SA) was also used for conjugation. SA was first transferred to the antibody before oxidized with periodate for conjugation to aminooxy-containing drug-linkers [47]. Several different antibody ADCs were prepared with two different drug-linkers. Anti-HER2 ADC prepared using this approach showed strong in vitro and in vivo antitumor activities. A similar method was developed by transferring C9-azido-modified SA into antibody for conjugation with DBCO containing doxorubicin using copper-free click chemistry [48]. Finally, there was a report showing the use of a mutant endoglycosidase in preparing site-specific ADC containing homogeneous glycans [49].

The glycoengineering approach is unique in that the drug-linkers were coupled to glycans without a need to engineer amino acid sequence and they are coupled far away from amino acid residues. It was demonstrated in a study as described above that the ADC made through glycoconjugation was more efficacious in vivo than conventional lysine conjugate. However, the method needs the special reagents and enzymes for glycoengineering.

## 5. Site-Specific ADC through Short Peptide Tags

There are several site-specific conjugation methods being developed through coupling of cytotoxin to specific short peptide tags that contain four to six amino acid residues.

Strop et al. engineered a glutamine tag (LLQG) into an antibody molecule, so the glutamine in the tag can be recognized by mTG for transferring amine containing drug [50]. Twelve out of 90 sites in an anti-EGFR antibody were found to be efficiently conjugated with mTG. They demonstrated that the drug-linker, MMAD, was efficiently transferred by the enzyme to the glutamine tags, including LLQGA in the C-terminal heavy chain or GGLLQGA in the C-terminal light chain. The ADCs generated were homogeneous with DAR at ~2 and they showed strong in vitro and in vivo antitumor activities. Interestingly, the ADC with the drug conjugated at the antibody light chain had better pharmacokinetics and serum stability in a species-dependent manner probably due to a different mechanism other than the chemical instability associated with cysteine conjugations.

Another conjugation platform was also reported by using sortase A-mediated transpeptidation reaction, generating ADCs with cytotoxins coupled to pre-defined sites [51]. This method includes C-terminal modification of antibody heavy and light chains with sortase A recognition motif, LPETG, which make them suitable substrates for sortase A-mediated transpeptidation of a pentaglycine peptide containing either MMAE or maytansine. Anti-CD30 ADC containing MMAE and anti-HER2 ADC containing maytansine were generated with sortase A from *S. aureus*. The ADCs displayed strong in vitro and in vivo antitumor activities similar to the ADCs generated using the conventional approaches.

Wu et al. described a site-specific antibody conjugation using a genetically encoded aldehyde tag [52,53]. A short peptide tag, LCxPxR, which was recognized by formylglycine-generating enzyme (FGE), was inserted into N or C terminal region of antibodies. After co-expression of the antibody with FGE, the cysteine in the short peptide tag was oxidized by the enzyme inside the cells to aldehyde-bearing formylglycine which can be coupled with aminooxy-functionalized reagents. Although the efficiency of conversion of cysteine to formylglycine varied among different locations of the inserted short peptide tag (44 to 91%), the site-specific conjugation was found to be efficient when the tag was introduced in either the N or C terminus of antibodies.

The methods in this category rely on introducing unique short peptide tags into antibodies for enzyme modification either in vivo or in vitro. They allow specific amino acids in the peptide tags to be functionalized and coupled to the drug-linkers. Although the approaches are straightforward, the potential immunogenicity of these short peptide tags located at different region of antibodies is currently unknown, nor the scalability.

## 6. Site-Specific Antibody Conjugation for Diagnosis

**Figure 3:**
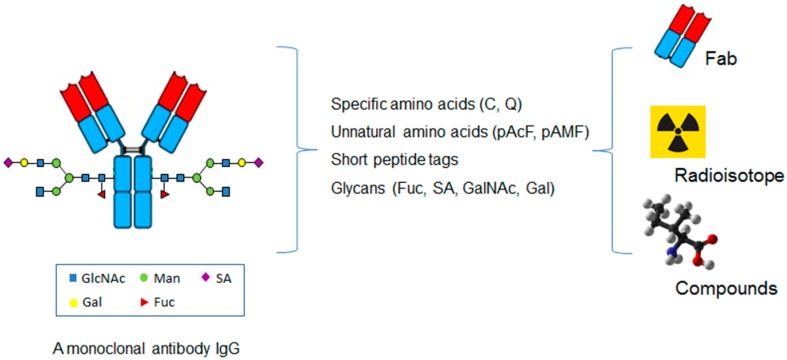
The application of the site-specific ADCs in coupling antibody with small protein, radioisotope, and non-cytotoxic compounds.

The recent technical advancements in positron emission tomography (PET) and optical imaging (OI) resulted in great interest in the development of multimodal PET/OI probes that can be employed during the diagnosis, staging, and surgical treatment of cancer [54]. The combination of PET/OI agents with antibodies using site-specific conjugations would enhance both the sensitivity and selectivity (Figure 3). A chemoenzymatic strategy for the construction of multimodal PET/OI and radiolabeled immunoconjugates was developed by the site-specific labeling of antibody N297 glycans [55,56,57]. The method includes the removal of terminal galactose, followed by enzymatic incorporation of azido-GalNAc using GalT (Y289L). Instead of being coupled to cytotoxins as described above, the azido sugar in the antibodies was conjugated with chelator- or chelator plus fluorophore–modified DBCO through SPAAC before the antibodies was radiolabeled. In one study, an anti-PSMA antibody was site-specifically conjugated with the chelator desferrioxamine-modified DBCO through antibody glycans using copper-free click chemistry [55]. The chelator-modified antibody was then radiolabeled with the position-emitting radiometal ^89^Zr. The radiolabeled antibody displayed high selective tumor uptake and tumor-to-background contrast in mice bearing PSMA expressing tumor. In another study, a colorectal cancer–targeting antibody was GalT (Y289L) modified with azido-GalNAc which was then conjugated with two reporters [56]. These reporters include DBCO-containing near-infrared fluorescent dye Alexa Fluor 680 and DBCO containing desferrioxamine which subsequently reacted with the positron-emitting radiometal ^89^Zr. In in vivo PET and fluorescence imaging experiments, a hybrid ^89^Zr- and Alexa Fluor 680-labeled antibody conjugate displayed high levels of specific uptake in the tumor in mouse xenograft models. The data suggest that the site-specific conjugation strategy is robust and reproducible, producing well-defined immunoconjugates.

Antibody was also site-specifically conjugated with PET, near-infrared fluorescent (NIRF), and dual-modal (PET/NIRF) imaging agents [57]. The N297 glycans of anti-CA19.9 (a tumor associated carbohydrate antigen) was remodeled and incorporated with azido-GalNAc using GalT (Y289L). The azido sugar was then coupled using copper-free click chemistry with DBCO-containing desferrioxamine for ^89^Zr radiolabeling for PET imaging used in noninvasive whole-body imaging and/or a NIRF dye for guided delineation of surgical margins. The antibodies conjugated with single or dual imaging agents showed specific uptake and contrast in antigen-positive tumors with negligible nonspecific uptake in antigen-negative tumor in a mouse xenograft model using human pancreatic cancer cell lines.

In another study, a colorectal cancer-targeting antibody was conjugated with trans-cyclooctene through N297 glycans [58]. The immunoconjugate was injected into human colorectal carcinoma xenografts after the administration of a pretargeted PET imaging agent, ^64^Cu-labeled tetrazine radioligand. The antibody and radioligand reacted in vivo in mice via strain-promoted azide-alkyne click chemistry. PET imaging and biodistribution studies revealed that this strategy clearly delineated tumor tissue, producing images with excellent contrast and high tumor-to-background ratio.

Kazane et al. developed a site-specific DNA-antibody conjugation method for specific and sensitive immuno-polymerase chain reaction (PCR) used as diagnosis and imaging [59]. Unnatural amino acid, pAcF, was introduced into anti-HER2 antibody Fab at LC-K169 or LC-S202 and it was coupled with aminooxy-modified ssDNA primer (32 nt) to produce oligobody. After antigen binding of the Fab, the oligonucleotide was amplified, ligated, and hybridized with complementary fluorophore which was detected with either a fluorescence or confocal microscope. The immunoconjugates were tested in immuno-PCR assays to detect HER2 expressing tumor cells. They showed greater sensitivity and specificity as well as a lower background signal than nonspecifically coupled fragments and can detect extremely rare tumor cells in a complex cellular environment. Thus, the site-specific antibody-oligonucleotide conjugates should provide sensitive and specific reagents for diagnostics.

The use of site-specific conjugation for radiolabeling or coupling of antibody with fluorescence/oligonucleotides could potentially improve sensitivity and specificity. It could reduce false positive outcome in cancer diagnosis. However, there is no in vivo data yet which demonstrated its superior selectivities as compared to the immunoconjugates prepared using conventional methods.

## 7. Site-Specific Antibody Conjugation for Other Therapeutic Applications

The site-specific conjugation approaches have also been applied to other small molecular weight proteins or compounds in addition to cytotoxins or radioisotopes (Figure 3) [60].

The antibody fragments have been coupled using site-specific method as bispecific antibody Fab conjugates. In a study, bispecific antibody Fab conjugate was generated using genetically encoded unnatural amino acids with orthogonal chemical reactivity as described above for cytotoxin containing ADC [61]. A tRNA/aaRS pair derived from *Methanococcus jannaschii* was co-expressed to site-specifically incorporate pAcF at defined sites in each of two Fab fragments in response to an amber nonsense codon. The unnatural amino acids was incorporated into LC-S202 of Fab fragment of anti-HER2 and subsequently conjugated with PEG linker containing azide, while pAcF was incorporated in HC-K138 of Fab fragment of anti-CD3 before conjugated with PEG linker containing BCN. Both Fab fragments were subsequently coupled using copper-free click chemistry. In an in vitro cytotoxicity assay, the bispecific antibody Fab conjugate efficiently recruited T cells from human peripheral blood mononuclear cells (PBMCs) to kill the target tumor cells at picomolar concentration. Moreover, the same site-specific antibody conjugation approach was applied to prepare bispecific antibody Fab conjugates against both CD3 and C-type lectin-like molecule-1 (CLL1) as well as both CD3 and CD33 [62]. CLL1 and CD33 are cell surface antigens overexpressed in acute myeloid leukemia, and the antibody Fab fragments were coupled to either azido-PEG3-aminooxy or BCN-PEG3-aminooxy linkers, respectively. The bispecific antibody Fab conjugate against CD3 and CLL1 displayed strong in vitro and in vivo antitumor activity as compared to the Fab conjugate against CD3 and CD33. In addition to use of PEG linker for coupling, either oligonucleotides or peptide nucleic acids of defined sequences were site-specifically conjugated to unnatural amino acids introduced in antibody for preparation of bispecific antibody Fab conjugates or multimeric antibody Fab conjugates [63]. As described above, pAcF was incorporated into HC-K138 of anti-CD3 Fab, while pAcF was introduced into LC-S202 of anti-HER2. Complementary peptide nucleic acid strands were then coupled to both Fab fragments, respectively. The bispecific Fab conjugates were self-assembled based on Waston-Crick base pairing properties of oligonucleotides. They were shown to recruit cytotoxic T cells to kill cancer cells in vitro. Tetrameric Fab conjugates were also generated using similar approach.

Besides the bispecific antibody Fab conjugation, chemically programmed bispecific antibody conjugation is another strategy for site-specifically preparing a bispecific antibody. Kim et al. reported the generation of a bispecific small molecule-antibody conjugate for targeting prostate cancer [64]. They incorporated an unnatural amino acid, pAcF, into different locations of anti-CD3 Fab that are distal to the antigen-binding site based on the crystal structure. The unnatural amino acid in the Fab was conjugated to a synthetic small molecule ligand, 2-[3-dicarboxy propyl]-ureido] pentanedioic acid (DUPA), that selectively binds PSMA with high affinity. A bivalent Fab was also prepared by introducing pAcF in two different positions (LC-S202 and HC-K138) and subsequently coupled with two DUPA ligands to a single Fab. The anti-CD3 DUPA conjugate showed potent in vitro cytotoxicity against prostate cancer cells and strong in vivo efficacy in mouse xenograft models. Cui et al. also reported another approach in preparing chemically programmed bispecific antibodies [65]. A C-terminal selenocysteine (Sec) was cotranslationally introduced into anti-CD3 antibody Fab. The Sec containing Fab was conjugated with maleimide containing LLP2A, a high affinity ligand for integrin α4β1 overexpressed in malignant B cells, or maleimide-containing folate, a high affinity ligand for folate receptor α overexpressed in many cancer cells. The bispecific small molecule-antibody conjugates showed potent and specific in vitro and ex vivo cytotoxicity against tumor cell lines and primary tumor cells in the presence of T cells. In another study, a diabody containing both anti-hapten and anti-CD3 Fv (disulfide linked polypeptides containing either variable heavy or variable light chains) was coupled with hapten-derivatized small molecule folate through a reactive lysine introduced in one of the polypeptide of anti-hapten [66]. The chemically programmed diabody demonstrated high selectivity and potency against folate receptor α-expressing ovarian cancer cells both in vitro and in vivo.

Lehar et al. reported generation of novel antibody-antibiotic conjugate (AAC) using THIOMAB approach [67]. They found a virulent subset of bacteria *Staphylococcus aureus* that can establish infection even in the presence of antibiotics vancomycin. They prepared an AAC in which an anti-*S. aureus* antibody was conjugated to a highly efficacious antibiotics rifalogue that is activated only after being released in lysosome. A cysteine residue was engineered at the V205 position of ant-bacteria antibody light chain and the thiol containing antibody was conjugated to MC-vc-PAB-rifalogue. The AAC is superior to vancomycin for the treatment of bacteremia in vivo.

A site-specific antibody-polymer conjugation (APC) approach was also reported [68]. An unnatural amino acid, pAcF, was introduced into anti-HER2 Fab (LC-S202) or IgG (HC-Q389) using an evolved orthogonal tRNA/aaRS pair. The engineered ketone-containing unnatural amino acid was conjugated with aminooxy-derivatized cationic block copolymer. The cationic polymer on the antibody specifically delivered siRNAs to HER2-positive tumor cells and mediated potent gene silencing at both the mRNA and protein levels in vitro.

The site-specific antibody conjugation has been applied for preparing immunoconjugates against other diseases such as autoimmune disease and atherosclerosis. A highly potent phosphodiesterase 4 (PDE4) inhibitor, GSK256066, was site-specifically coupled to a human anti-CD11a through unnatural amino acid, pAcF, introduced at HC-A122 with DAR at ~2 [69]. PDE4 is a cAMP phosphodiesterase widely expressed in variety of cells and some small molecule PDE4 inhibitors showed wide-ranging preclinical efficacy in autoimmune diseases with a few being approved for the treatment of some moderate to severe inflammatory conditions. However, dose-limiting side effects have impeded their broader therapeutic application. The site-specific conjugation of pan-immune cell targeting human anti-CD11a with GSK256066 resulted in an immunoconjugate that rapidly internalized into immune cells and suppressed lipopolysaccharide (LPS)-induced TNFα secretion in primary human monocytes. In another study, a liver X receptor (LXR) agonist was site-specifically conjugated to pAcF at HC-A122 of anti-CD11a [70]. Liver X receptor agonists have been explored as potential treatments for atherosclerosis and other diseases based on their ability to induce reverse cholesterol transport and suppress inflammation. However, this therapeutic potential has been limited by on-target adverse effects in the liver mediated by excessive lipogenesis after the interaction of the ligand with LXR-α. To prevent the adverse effect, the aminoooxy-modified LXR agonist was coupled to pAcF in anti-CD11a for selective delivery of the agonist to monocytes/macrophages while sparing hepatocytes. The anti-CD11a IgG-LXR agonist immunoconjugate induced LXR activation specifically in human THP-1 monocyte/macrophage cells in vitro with EC_50_ at nM range, but had no significant effect in hepatocytes, indicating that payload delivery was CD11a-mediated. This approach represents a fundamentally different strategy that uses tissue targeting to overcome the limitations of LXR agonists for potential use in treating atherosclerosis.

As next generation technologies, the site-specific antibody conjugations are likely to be applied to different therapeutic areas for preparing homogeneous immunoconjugates. The combination of therapeutic antibodies with wide varieties of small molecular weight proteins or drugs would potentially expand the current treatment options for many challenging diseases. Although it is unknown which methods are better than others, each category of these methods may have their unique advantages related to the in vivo and in vitro properties of the conjugates.

## 8. Conclusions

Site-specific antibody drug conjugation technologies have been developed by coupling the cytotoxin to engineered specific amino acids, unnatural amino acids, short peptide tags, and N297 glycans. As next generation methods, these approaches generated homogeneous ADCs with a high therapeutic index as compared to the conventional conjugations. Some of the site-specific ADCs even showed better antitumor efficacy in vivo than the ADCs prepared using conventional methods. Moreover, there are trends in applying these site-specific antibody conjugations to other therapeutic areas or diagnosis. All those studies have provided promising results that suggest the usefulness of these next generation methods in the coupling of small proteins, small molecular weight compounds, DNAs, and RNAs. It is not surprising that these new technologies will lead to important therapeutic platforms for many unmet medical needs.

## Figures and Tables

**Table 1:** The four categories of the site-specific antibody-drug conjugate (ADC).

Technologiees	Specific Amino Acids	Unnatural Amino Acids	Glycans	Short Peptide Tags
Conjugation sites	C, Q	pAcF, pAMF, Sec etc.	SA, Gal, Fuc etc.	LLQG, LCxPxR etc.
Cell line engineering	−	+	−	±
Metabolic labeling	−	+	±	−
In vivo protein engineering	+	+	−	+
In vitro enzymatic modification	±	±	+	+
Chemical modification	+	−	±	−
Selected references	[27,28,29,30,31,32,33,34,35]	[36,37,38,39,40,41]	[42,43,44,45,46,47,48,49]	[50,51,52,53]

## Abbreviations

ADC	Antibody-drug conjugates
AML	Acute myelogenous leukemia
ALL	Acute lymphoblastic leukemia
MMAE	Monomethyl auristatin E
PBD	Pyrrolobenzodiazepine
LC	Light chain
mTG	Microbial transglutaminase
SPAAC	Strain-promoted azide-alkyne cycloaddition
aaRS	Aminoacyl-tRNA synthetase
EGFR	Epidermal growth factor receptor
pylRS	Pyrrolysyl-tRNA synthetase
pAMF	*p*-azidomethyl-l-phenylalanine
DBCO-PEG-MMAF	DBCO-PEG-monomethyl auristatin F
Sec	Selenocysteine
GlaNAc	*N*-acetylgalactosamine
BCN	Bicyclononyne
FGE	Formylglycine generating enzyme
OI	Optical imaging
NIRF	Near-infrared fluorescent
PBMCs	Peripheral blood mononuclear cells
DUPA	2-[3-dicarboxy propyl]-ureido] pentanedioic acid
APC	Antibody-polymer conjugation
LPS	Lipopolysaccharide
IgG	Immunoglobulin G
HER2	Human epidermal growth factor receptor 2
DAR	Drug-to-antibody ratio
DM1	Mertansine
HC	Heavy chain
DBM	Dibromomaleimide
DBCO	Dibenzocyclooctynes
pAcF	*p*-acetylphenylalanine
CHO	Chinese hamster ovary
MMAD	Monomethyl auristatin D
tRNA pyl	Pyrrolysyl-tRNA
TyrRS	Tyrosyl tRNA synthetase
MMAF	Monomethyl auristatin F
SECIS	Sec incorporation sequence
GlcNAc	*N*-acetylglucosamine
SA	Sialic acid
PET	Position emission tomography
PSMA	Prostate specific membrane antigen
PCR	Polymerase chain reaction
CLL1	C-type lectin-like molecule-1
AAC	Antibody-antibiotic conjugate
PDE4	phosphodiesterase 4
LXR	Liver X receptor
